# PGMS: A Case Study of Collecting PDA-Based Geo-Tagged Malaria-Related Survey Data

**DOI:** 10.4269/ajtmh.13-0652

**Published:** 2014-09-03

**Authors:** Ying Zhou, Neil F. Lobo, Adam Wolkon, John E. Gimnig, Alpha Malishee, Jennifer Stevenson, Sulistyawati, Frank H. Collins, Greg Madey

**Affiliations:** Department of Computer Science and Engineering, University of Notre Dame, Notre Dame, Indiana; Department of Biological Sciences, University of Notre Dame, Notre Dame, Indiana; Centers for Disease Control and Prevention, Atlanta, Georgia; Ifakara Health Institute, Dar es Salaam, Tanzania; Department of Disease Control, London School of Hygiene and Tropical Medicine, Keppel Street, London, United Kingdom; Department of Public Health, University of Ahmad Dahlan, Yogyakarta, Indonesia

## Abstract

Using mobile devices, such as personal digital assistants (PDAs), smartphones, tablet computers, etc., to electronically collect malaria-related field data is the way for the field questionnaires in the future. This case study seeks to design a generic survey framework PDA-based geo-tagged malaria-related data collection tool (PGMS) that can be used not only for large-scale community-level geo-tagged electronic malaria-related surveys, but also for a wide variety of electronic data collections of other infectious diseases. The framework includes two parts: the database designed for subsequent cross-sectional data analysis and the customized programs for the six study sites (two in Kenya, three in Indonesia, and one in Tanzania). In addition to the framework development, we also present our methods used when configuring and deploying the PDAs to 1) reduce data entry errors, 2) conserve battery power, 3) field install the programs onto dozens of handheld devices, 4) translate electronic questionnaires into local languages, 5) prevent data loss, and 6) transfer data from PDAs to computers for future analysis and storage. Since 2008, PGMS has successfully accomplished quite a few surveys that recorded 10,871 compounds and households, 52,126 persons, and 17,100 bed nets from the six sites. These numbers are still growing.

## Introduction

The Malaria Indicator Survey (MIS) Basic Documentation for Survey Design and Implementation was developed by the Roll Back Malaria (RBM) Monitoring and Evaluation Reference Group (MERG) Household Survey Task Force.^1^ This documentation is comprehensive, including a set of guidelines, questionnaires, tabulations, and relevant manuals to guide how large-scale household-level surveys should be conducted. The malaria-related questionnaires on which the personal digital assistant (PDA)-based Geo-tagged malaria-related data collection tool (PGMS) is tailored from the previous package and implemented by the Bill and Melinda Gates Foundation sponsored Malaria Transmission Consortium (MTC)^2^ specifically for its six study sites, including the highlands and lowlands of Kenya, three low-to-moderate-to-high level transmission areas of Purworejo, Lampung, and Halmahera in Indonesia, and Dar es Salaam in Tanzania. The data collected by PGMS in collaboration with other entomological, parasitological, serological data will be used for the assessment of malaria epidemiology in a wide range of malaria transmission level sites, for the monitoring and evaluation of malaria control strategies, and for the development and calibration of malaria epidemiological models. As a result of the specific malaria research goals of MTC, the questionnaires presented in this work is not the full MIS, because it does not include a women's questionnaire; knowledge, attitude, and practices (KAP) questions, etc. Furthermore, unlike the MIS, the questionnaires are not national and were only conducted on the six study sites.

It is well known that implementing traditional large-scale paper-based household surveys in the developing world is costly and time-consuming, and presents considerable reliability and logistical difficulties. However, surveys must be conducted periodically because they collect valuable field data to help measure indicators related to disease prevalence and intervention monitoring and assessment. As mobile devices such as PDAs, global positioning system (GPS) units, GPS-enabled smartphones, etc., have become cheaper and ubiquitous, they have been increasingly used for various health surveys in the field.^3^–^18^ This is especially true for the surveys conducted in developing countries.

Ali and others^15^ used PDAs to finish a large survey enumeration and mass vaccination campaign, and they observed that people with little education in the use of computers had no difficulty in using mobile devices, which was also found by Rajput and others^16^ when they used mobile phones to perform clinical care during home visits, and by Dwolatzky and others^5^ when they evaluated the feasibility of using GPS-linked PDAs to support tuberculosis control in South Africa. Forster and others^4^ developed a portable computer-assisted field data collection system, and found it had significantly fewer errors and required less time than the paper-based questionnaire. The lower error rate in a PDA-based survey has also been reported by Byass and others.^3^ In References 15 and 16, field researchers point out that the methodology of electronic data collection can provide rapid data summaries and facilitate the performance of initial data analysis. Zhang and others^19^ using smartphones and Fletcher and others^6^ using PDAs, respectively, compared the handheld computer-based surveys and the paper-based surveys in their field study, and both concluded that handheld devices are a feasible alternative to paper-based forms for the field data collection. Aanensen and others^20^ used smartphones to conduct surveys and submit data to a central database in the field successfully. Parker and others^21^ reported that use of a tablet-based version of the survey can more effectively recruit reluctant responders and have a higher survey response rate. Because tablet computers that have good usability and simplicity, longer battery life, and lower weight only appeared in the last 2 years, tablets were used by relatively fewer researchers in their field data collections when compared with PDAs and smartphones. Walther and others^22^ compared the conversional paper-based approach with four electronic data capture methods, respectively, using netbooks, PDAs, tablet computers, and telephone interview by mobile phone, and found that error rates for the tablet computers and netbooks are lower than those for PDAs and smartphones. Because of the strengths of tablets, such as bigger screens and longer life battery than PDAs and mobile phones, we believe more tablet computers will be adopted for electronic data collection in the future. In short, electronically collecting field data is affordable, providing improvements in quality, reliability, and efficiency for surveys conducted in remote and harsh environmental conditions.

Similar to the previous applications, the strengths of the mobile device-based system to collect malaria-related data are clear and summarized as follows:

- Mobile devices can store more information and are not constrained by the space limitations of paper-based questionnaires.
- Programs may yield higher quality and more reliable results than a paper-based survey. They cannot only help check for illegal or inconsistent data, but also incorporate survey skip patterns, which not only substantially reduces the data entry time, but also minimizes errors caused by field workers as a result of a lack of survey conducting experience.
- Data on mobile devices can be easily transferred to a relational database such as Microsoft Access, which makes it easy to check the data in the field so that lost or incomplete records can be revealed and recollected.
- A one-time investment in mobile device hardware and software eliminates many repeated costs for printing, data entry, and data cleaning. This is especially true for large-scale surveys.
- Mobile devices can be locked down by software for the purpose of data security for meeting research ethics guidelines, therefore only authorized persons can look at the data files.
- Mobile devices are portable and much lighter than carrying lots of paper questionnaires, and more resistant to rain, mud, etc.

Our project started in 2008, when cost-effective tablet computers were not available in the commercial market, and we did not need mobile devices to wirelessly transfer field data to our data center. Therefore, we chose PDAs running Windows Mobile as the mobile devices for our cross-sectional malaria-related surveys. Choosing the kind of mobile device is determined by several factors such as which device is able to meet the practical needs of a particular data collection, the costs, the availability in the current market, etc. In addition to PDAs, the other alternatives such as smartphones, tablet computers, netbooks, etc., can also be used to conduct surveys in the field.

This work presents a complete case study to electronically collect malaria-related data in MTC's six study sites. It emphasizes the framework of the data collection applications with details such as database design, program development, and survey development topics that were discussed in previous work^3^–^19^, but not explored in-depth. The PGMS presented in this work includes two parts: the database designed for subsequent cross-sectional data analysis and the programs customized for the six study sites. In addition to the framework development, we also present our methods used when configuring and deploying the PDAs to 1) decrease data entry errors, 2) conserve battery power, 3) field install the programs onto dozens of handheld devices, 4) translate electronic questionnaires into local languages, 5) prevent data loss, and 6) transfer data from PDAs to computers for future analysis and storage. In addition, we designed PGMS as a generic survey framework that can be used not only for malaria-related survey, but also for other similar infectious disease surveys involving households and people. The Centers for Disease Control and Prevention (CDC) adopted PGMS and have collected data in its parasitemia and anemia surveys since 2010. We successfully addressed two significant challenges of deploying PDA-based surveys in the field: how to prevent the accidental loss of valuable data and how to make data entry easier for users to guarantee data accuracy. The experience we learned from our project can also be used by smartphone-based, tablet-based, or netbook-based surveys. The design principles discussed in the Design and Implementation section can be applicable to a wide variety of data collection implementations, particularly those who might also be interested in implementing similar large-scale community-based geo-tagged electronic data collections.

## Design and Implementation

The PGMS takes a randomly sampled household list stored in a text file as an input. The list is generated by GPS Sample 2.0 (GPS2) developed at CDC.^23^ Interviewers use PGMS to conduct interviews for the households on the list.

### Hardware and software used in developing and conducting PGMS.

We used three brands of PDAs in the project: Hewlett- Packard iPAQ 210 Series (Palo Alto, CA), Socket SoMo Model 650-M (Newark, CA), and Dell Axims (×50 and ×51) (Round Rock, TX). The GPS units are GlobalSat BC-337 Compact Flash Series (Taipei, Taiwan). The OtterBox 3600 Cases (Fort Collins, CO) are used to protect PDAs from water, dust, and crushing. During rainy conditions, they still allow interviewers to operate the screens of the PDAs by membranes on the front. Other equipment includes charging cables, stylus spares, secure digital (SD) cards, car battery charging ports and cables, multisocket plugs, and similar components.

A few electronic questionnaire development tools were available, such as the Microsoft .NET Compact Framework, Census and Survey Processing System (CSPro),^24^ Visual CE, etc. The reason we finally chose Visual CE 10.4 Professional (Cambridge, MA)^25^ as the development tool is that we have gained lots of Visual CE programming experience from our previous development of electronic surveys.^23^ We also found that after our field workers who have some programming experience or related knowledge obtained additional quick training, they can now quickly make minor changes (such as adding new questions or deleting old questions in a form) on programs in the field where our program developer might not be available. In addition, the interface for questionnaire forms developed by Visual CE is user-friendly. After 1- or 2-day intensive training sessions on field data collection, field workers without PDA experience, we find, have no difficulty in conducting PDA-based questionnaires. Visual CE can only run Windows Mobile system. Microsoft has said that Windows Mobile 6.x support for customers ends in 2013. If Windows-Mobile devices are no longer on the market in the future, all forms developed by Visual CE will have to be converted to DroidDB^26^ forms that can run on Android devices.

Microsoft ActiveSync^27^ is used to synchronize data between a PDA and a PC and to install applications on PDAs. It is only for Windows XP and earlier versions of Windows, that have been replaced by Microsoft Windows Mobile Device Center for Windows Vista and Microsoft Windows 7.^28^ Pocket Controller^29^ is used to display the screen of the demonstrative PDA in real time on a projection system to assist in conducting data collection in training sessions. Sprite Clone helps with mass installation of PGMS on PDAs. Visual CE add-on tools is adopted to synchronize data between PDAs and PCs.

### Database design for cross-sectional data analysis.

One goal of our database design is to simplify the subsequent cross-sectional analysis on the data collected from the six study sites. To achieve this goal, the data type of most database fields is an integer rather than text so that the responses to most survey questions are saved as digital numbers in the tables. For example, whether a survey questionnaire is displayed to interviewers in English, Indonesian, or Kiswahili, the responses are stored as integers in the database independent of the languages displaying the survey questions.

We also designed two fields respectively called Expired and TimeStamp that allow us to track any operations (add, edit, or delete) performed by interviewers on the data records. Therefore, the particular activities for each interviewer on a given day can be reconstructed. For example, when an interviewer starts a questionnaire for a subject, the time is automatically saved into the TimeStamp field of the subject's newly created record in the system. The Expired field of the record is set as “N” to mean the record is valid (not expired). Later, if the interviewer finds the subject's medication history is not correctly recorded and needs to edit the existing subject's record, a new record is created with a newer time stamp, but the old one is not overwritten by the new one. It is instead marked as invalid by means of setting its Expired field as “Y.” Similar to the edit operation, deleting a record does not remove the record from the PDA physically, but just marks the record as invalid by filling in Expired with “Y.” Therefore, although the interviewer thinks he/she has deleted the record, it is actually still on the PDA and only becomes invisible to the interviewer. The Expired construct was inspired by Anatoly S. Frolov's method used in an electronic MIS questionnaire developed by Visual Studio.NET Compact Framework at the CDC+.^11^–^13^

**Figure 1.**
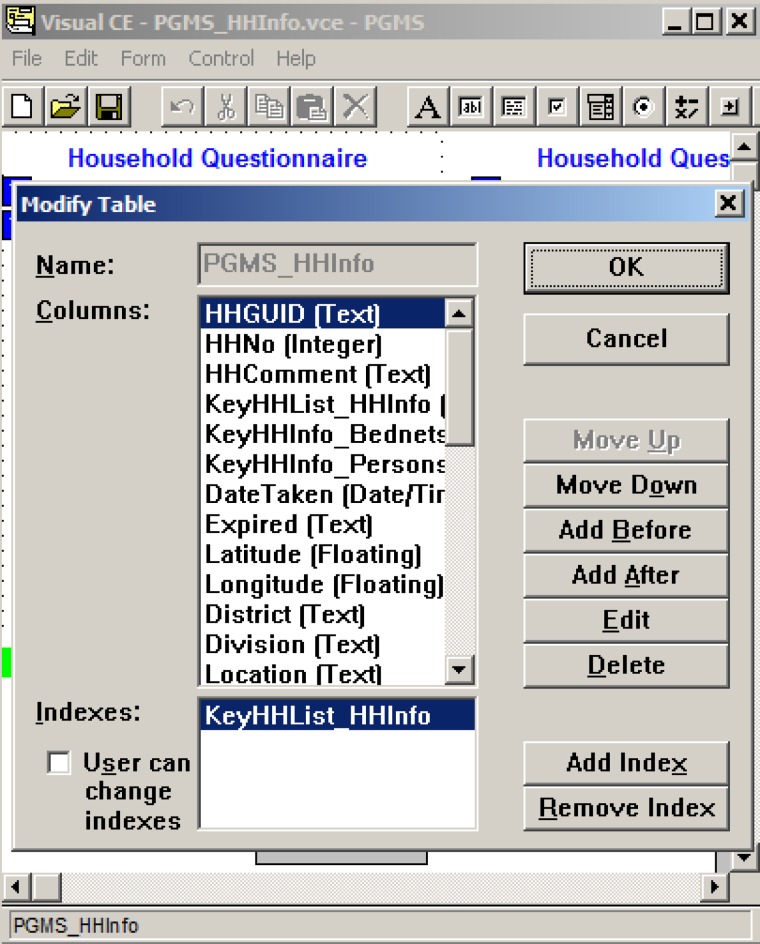
Using the Visual CE development environment to create and modify the fields in a table.

In Figure 1, we present an example to add, delete, and modify the fields (columns) in a table in the Visual CE development environment. A more detailed description of the fields in the seven tables can be found in the Supplement of Excel E1.

### Software design and customization for different study sites.

#### Outline of PGMS.

**Figure 2.**
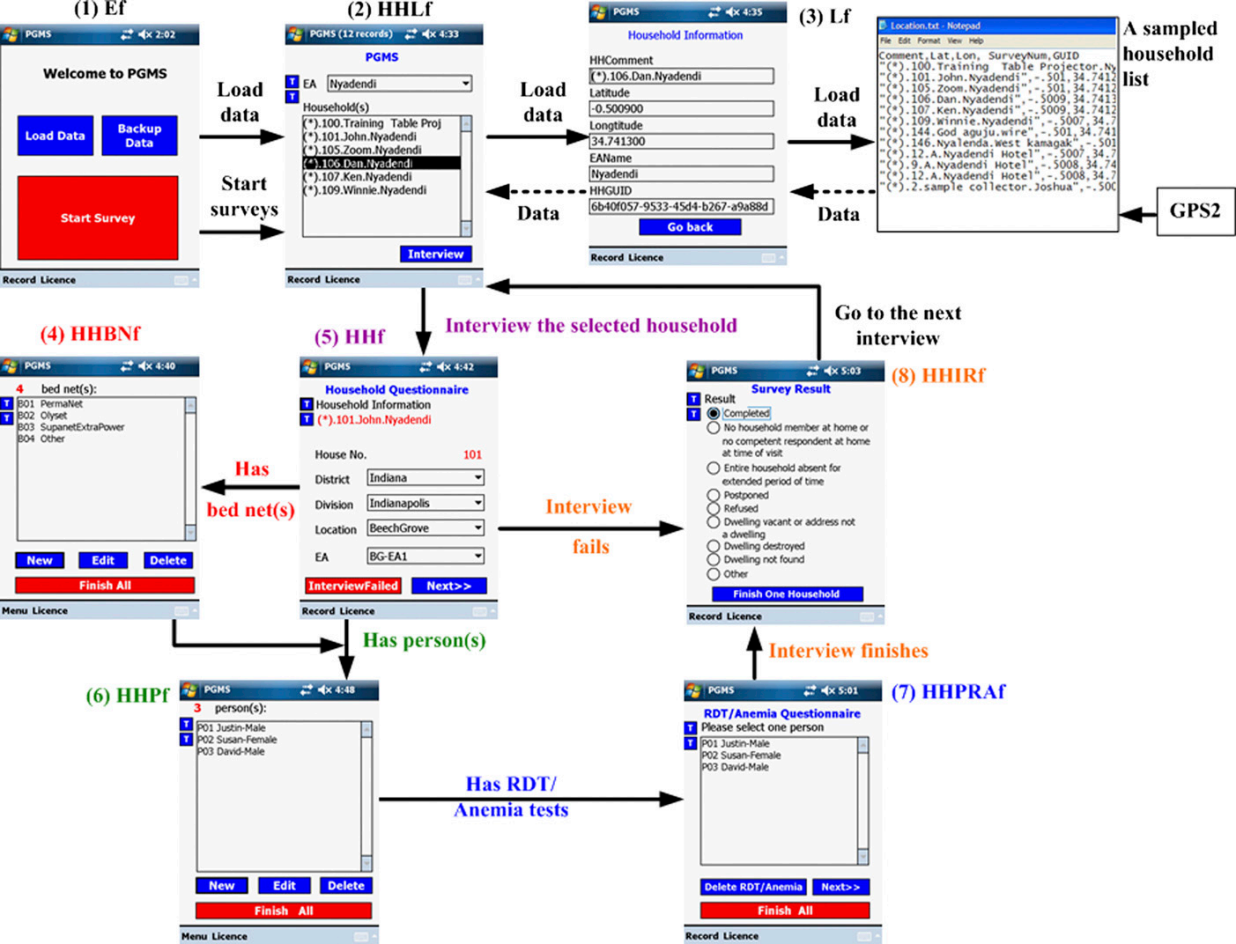
Overview of personal digital assistant (PDA)-Geo-tagged malaria-related data collection tool (PGMS) and the workflow of conducting a complete household-based interview. The survey typically consists of eight parts with the optional input of data from GPS2. 1) (Ef): the entry form where interviewers can load data, back up collected data to the secure digital (SD) card, and start surveys; 2) HHLf: the household listing form where interviewers can select one household and start a survey of the household; 3) Lf: the data-loading form that is called by the entry form to load data and runs at the backend of the system; 4) HHBNf: the bed net form where interviewers record the information of all bed nets used in the household; 5) HHf: the household form where interviewers can enter the basic house-related information for the selected household such as roof and floor condition; 6) HHPf: the person form where interviewers enter the interviewee's basic information and malaria and medication history; 7) HHPRAf: the RDT-anemia form where interviewers record the results of two blood tests for each interviewee; 8) HHIRf: the interview result form where the survey result such as “completed” is recorded.

Here, as an example, we discuss a survey using the house as the sampling unit. Figure 2 shows the survey typically consists of eight parts. The arrows show how to conduct a complete household-based interview by using PGMS (see Multimedia S1 for more details of the process).

When an interviewer launches PGMS on a PDA, the software entry form (Ef) first pops up where the interviewer is able to do three things: 1) load data from an input file to the house- hold listing form (HHLf) by calling the data loading form (Lf) at the backend of the system; 2) backup data from the PDA to the SD card; 3) start a survey for the selected household. Data loading is a one-time operation and happens upon first use of the PDA to conduct interviews.

After the data loads, the interviewer clicks on the “Start- Survey” button to start the surveys for the households shown in the input file.

He/she is then led to HHLf, where the dropdown list has already been populated by the previous loading process with the names of the enumeration areas (EAs) (villages). When an EA is chosen (see “Nyadendi” on HHLf shown in Figure 2), HHLf will display the corresponding households in the selected EA.

After the interviewer selects one household (see “(*).105.Zoom.Nyadendi” on HHLf shown in Figure 2) and clicks on the “Interview” button on HHLf, he/she will be taken to the household form (HHf) to enter the basic house information for the selected household. If the household has bed net(s), the interviewer will be guided from HHf to the bed net form (HHBNf) after he/she finishes entering the house information; otherwise, he/she will go directly to the person form (HHPf) to enter the interviewee's basic information and malaria and medication history. Afterward, the interviewer records the results of two blood tests for each person shown during the survey on the rapid diagnostic test (RDT)-anemia form (HHPRAf).

Finally, to complete one survey, the interviewer chooses the survey result such as “completed,” “postponed,” “refused,” or other reasons on the interview result form (HHIRf).

Currently, PGMS used in the lowlands and highlands of Kenya has an extra part: the compound form. The sampling unit in these areas is the compound, which is generally made up of two or more houses. As a result, the architecture of PGMS shown in Figure 2 includes the compound form as an extra layer between HHLf and HHf (not shown here).

**Figure 3.**
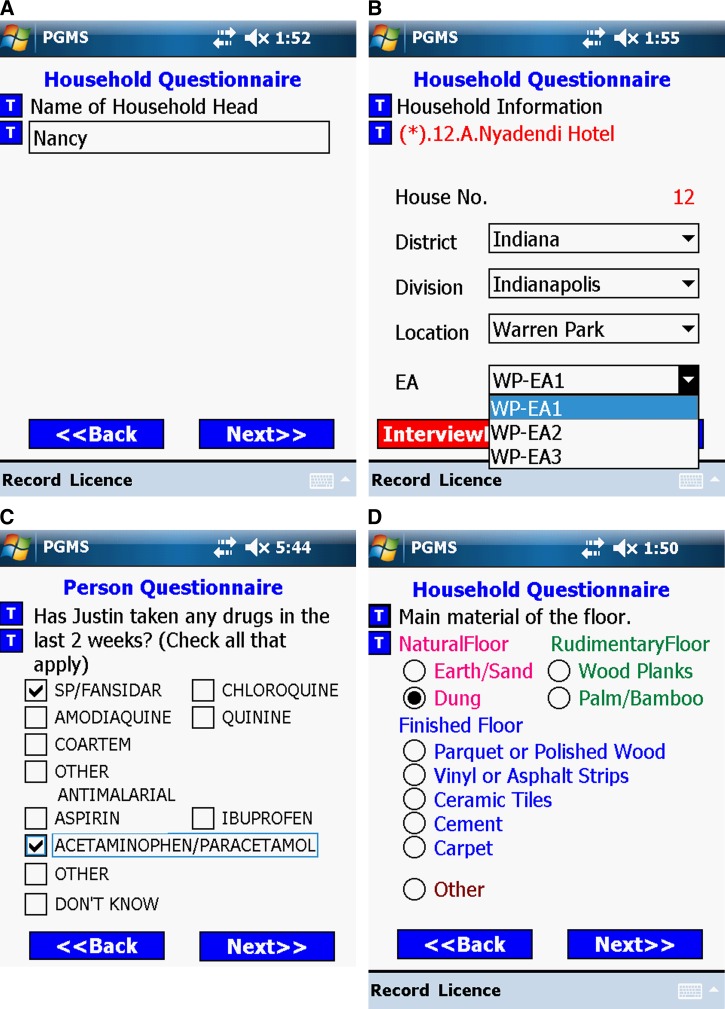
Four kinds of controls used in personal digital assistant (PDA)-Geo-tagged malaria-related data collection tool (PGMS). (**A**) Text entry, (**B**) Dropdown lists, (**C**) Checkboxes, and (**D**) Radio buttons.

Four major controls are used to enter data into PGMS: radio buttons, checkboxes, dropdown lists, and text entry fields. To avoid data entry errors, we choose different controls for different entry scenarios. According to the feedback obtained in training sessions, we compared the ease of use of the four controls and found text entry fields to be least efficient and most error prone. Therefore, we use text entry fields as little as possible; they are only used when a number of unknown characters need to be input, such as names and some numeric entries (Figure 3A). Dropdown lists and checkboxes allow users to select multiple items from a list of possible choices. Checkboxes enable clearer layout to elicit faster performance (see Figure 3C) and are preferred over dropdown lists. When one screen cannot accommodate enough checkboxes to display all responses for one question, we have to adopt dropdown lists. Visual CE 10 (the development tool of PGMS that is described in the Hardware and Software Used in Developing and Conducting PGMS section in more detail) also provides a kind of special dropdown list called a dependent dropdown list that automatically filters the options in a dependent dropdown list according to the value that the user selects in another control on the form. For example, in Figure 3B, Location is the filter that the options shown in the EA dropdown list depend on. Under the location of E.NYAKACH, there are only two EAs: DOL/NDUGA and KAMWANA. We provide radio buttons rather than dropdown lists when users need to choose one response from a list of mutually exclusive options because radio buttons can provide a more friendly user interface (Figure 3D). To prevent controls from becoming “invisible” under the strong sunshine conditions, which are very common in tropical areas, we use + on a light PDA background to produce a high contrast.

For the sake of cross-sectional data analysis, PGMS was developed to incorporate a core questionnaire that is conducted at all study sites. After finishing the development, we send PGMS to each site where local researchers help test all programs. According to the feedback from each site, we amend PGMS on the basis of the core questionnaire, such as adding extra survey questions or changing the order of questions, and send the updated version back to the site. This back-and-forth process of testing and amending for each site usually takes 2–3 weeks before a real survey starts at the site.

#### Design of comprehensive outline checks and cross-references between different forms.

**Figure 4.**
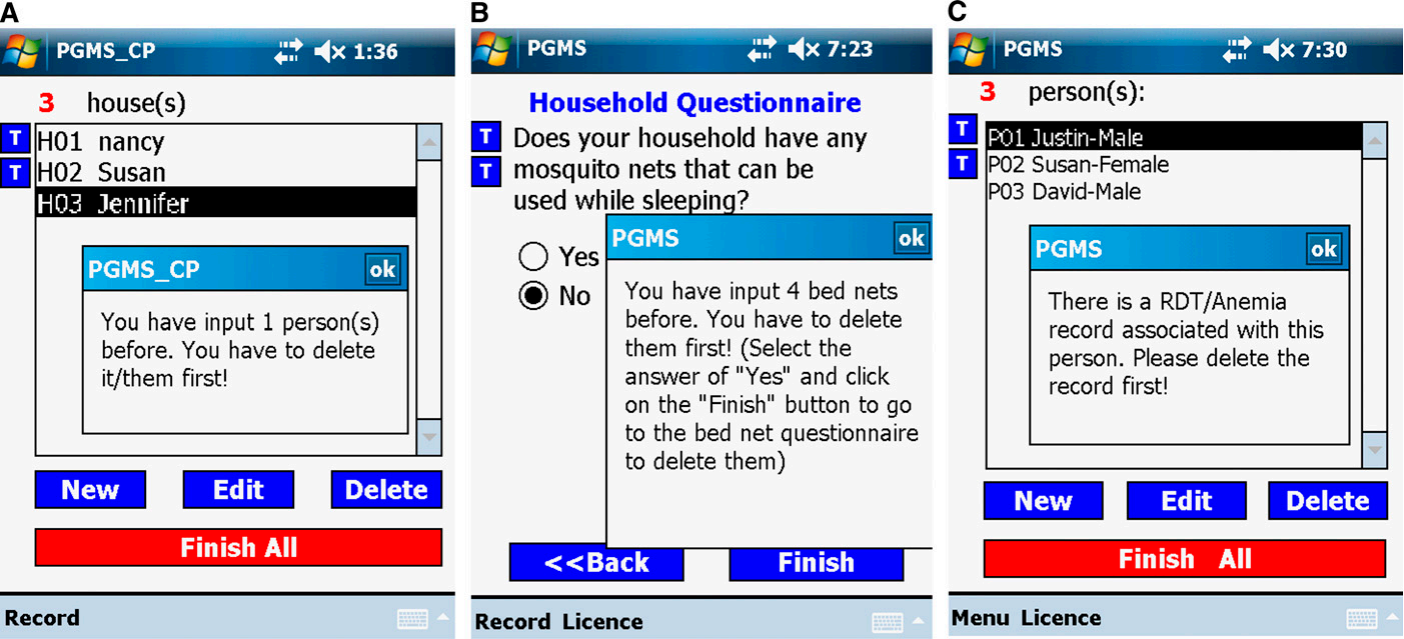
Three examples of comprehensive outline checks. (**A**) Outline check among the household table, the bed net table, and the person table. (**B**) Outline check between the household table and the bed net table. (**C**) Outline check between the person table and the rapid diagnostic test (RDT)-anemia table.

Except for simple entry data range checks, such as person age check, PGMS also implements several comprehensive outline checks. In the system with the compound as the sampling unit, PGMS does not allow users to delete a household record if its related bed net records or/and people records have not been deleted (Figure 4A). When the interviewer edits the household information questionnaire, if he/she input bed net records in the bed net questionnaire of this household previously, he/she is not allowed to answer “No” to such a household question as “Does your household have any mosquito nets that can be used while sleeping?” (Figure 4B). This outline check was implemented by PGMS checking on the bed net table and returning the number of bed net records of that household to the household form. As shown in Figure 4C, if an interviewee has a related RDT record in the RDT-anemia table, the interviewee's personal information record is not allowed to delete to keep the data consistency in the database. Therefore, if we want to remove a person's record, we have to go to the RDT-anemia form to delete his/her associated RDT-anemia record first, and then go back to the person form to delete the person record. All of these outline checks involve cross-reference to at least two forms.

**Figure 5.**
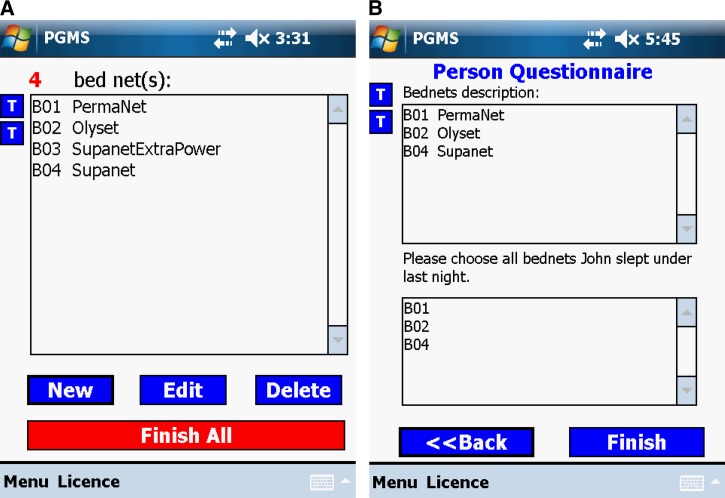
One example of displaying records from a related table to the interviewer.

Some questions in our malaria-related surveys require that a form is able to display records from a related table to the interviewer. For example, in the person questionnaire, each interviewee needs to answer which bed net he/she slept under the night before the survey was conducted. Figure 5 shows all entered bed nets in this household. In Figure 5, the bed net dropdown list in the person's questionnaire shows all bed nets that were hung up the night before the survey was conducted. The person form retrieves all qualified bed nets from the bed net table, screening not hung-up bed net(s) (It is “B03 Su- panetExtraPower” in this example.).

### Translating electronic questionnaires in local languages.

Because the ultimate operators of the survey programs are field workers and many do not know English, translating electronic questionnaires into local languages is a practical task we have to handle in the field. In this project, PGMS is translated in four languages (Bahasa [Indonesia], Kiswahili [highlands and lowlands of Kenya and Tanzania], DhoLuo [lowlands of Kenya], and Kisii [lowlands of Kenya]). The translations are done in three ways.

**Figure 6.**
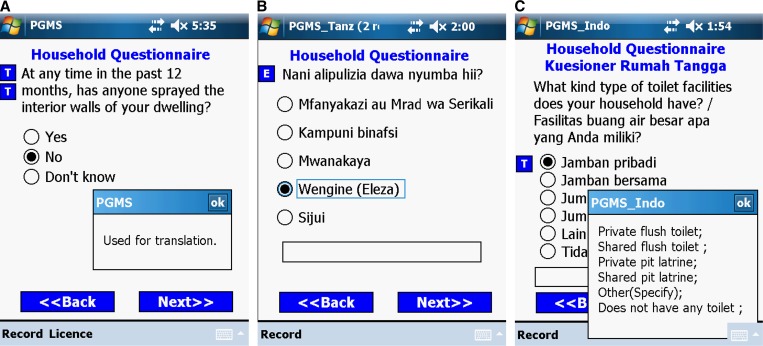
Three ways to translate electronic questionnaires into local languages. (**A**) Message box gives local language translation. (**B**) Questionnaire displayed in one local language. (**C**) Questionnaire presented simultaneously in English and Indonesian.

The first method shown in Figure 6A involves placing a command button beside each question. When the user clicks on the command button the local language translation pops up in a message box. This method is straightforward and the programs are easy to maintain. Moreover, people in the field can very easily change the translations in the text box if they are incorrect. The drawback to this method is that the responses to each question are not translated. This method is adopted by the highlands of Kenya, where the local staff is fluent in English and all local languages.

The second method, Figure 6B, involves maintaining two versions of the programs for the same questionnaire. One is developed in English by a programmer. Based on the English version, a field technician who knows the local language and who has some programming experience then replaces the English texts on all forms with the local language. As a result, the user interface is very friendly because all survey questions and responses are shown in a single language. However, it is tedious to maintain two versions of the programs, moreover inconsistencies can also occur. This second method is used in the questionnaires of Tanzania and the lowlands of Kenya.

The last approach (Figure 6C), used in Indonesia, presents each survey question and its responses simultaneously in English and in Indonesian on the same screen. Unfortunately, if one screen is not big enough to display all information in both languages, we revert to the first method and use command buttons.

### Survey data storage and synchronization between PDAs and PCs.

Because first-hand data collected in the field is very precious, we have taken three measures to avoid accidental loss of raw survey data. First, we ask interviewers to save data from PDAs to SD cards frequently during the day. To encourage them to do this, we place the backup command button on the Ef form so that interviewers can conveniently backup the existing data on PDAs to SD cards whenever they start new surveys. The second measure is that SD cards must not be cleared unless a cluster is completed. Only supervisors are authorized to clear data on SD cards; this procedure is implemented by adding password protection to SD cards on the PDAs. The final measure we take is to create several folders on a PC (the number of them depends on the number of PDAs the site is using). Within each folder, subfolders are created and named according to the dates when the data is collected. For example, the raw data file from PDA1 used in Lampung in Indonesia at the end of the day July 1, 2010 would be placed into the folder of \Indonesia\Lampung PDA1\07012010 on the PC. Therefore, at the end of each survey day, every raw data file actually stores the data collected before and on that day. Even if an entire PDA unit is lost on a given day, we only lose the data that is collected on that day. The data collected before the day the PDA is lost has already been saved on the PC.

**Figure 7.**
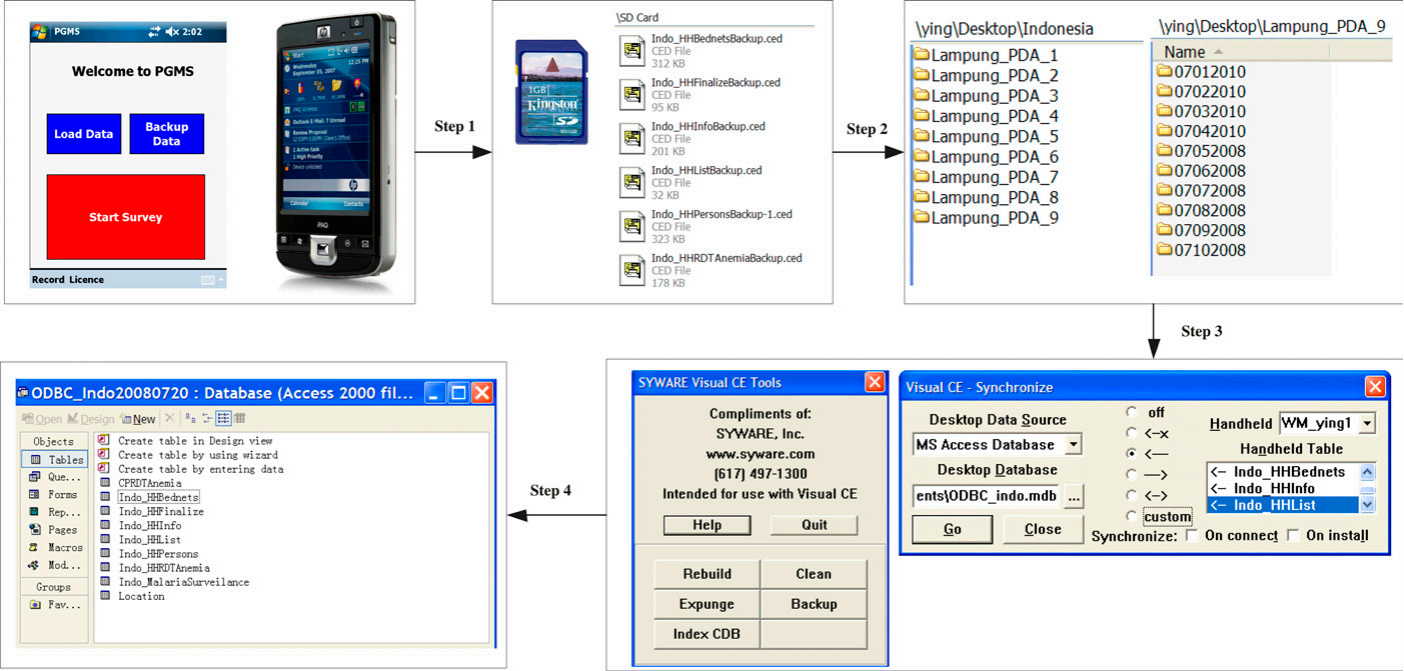
The four steps to dump questionnaire data from personal digital assistants (PDAs) to the Microsoft Access database.

The free SYWARE Visual CE add-on tools^30^ and the synchronization function built in Visual CE 10^25^ development environment are used to dump data from the raw data files into a Microsoft Access database. The steps of dumping data from PDAs to the Access database are summarized in Figure 7. In step 1, interviewers use the Backup functionality on the Ef form of PGMS to back up the collected data from PDAs to SD cards. Next, supervisors or data managers copy these data files, which end with the .ced extension, from the SD cards to the corresponding folders they create in advance (step 2) on their computers. In step 3 and step 4, the data from the data files is merged by Visual CE add-on tools and synchronized to the Access database by the synchronization function in Visual CE 10. Each step is described in more detail in Text S2.

### Preparation of PDAs for cross-sectional surveys.

This section describes how we customized the PDAs and installed our application to make them ready to use for cross-sectional questionnaires.

**Figure 8.**
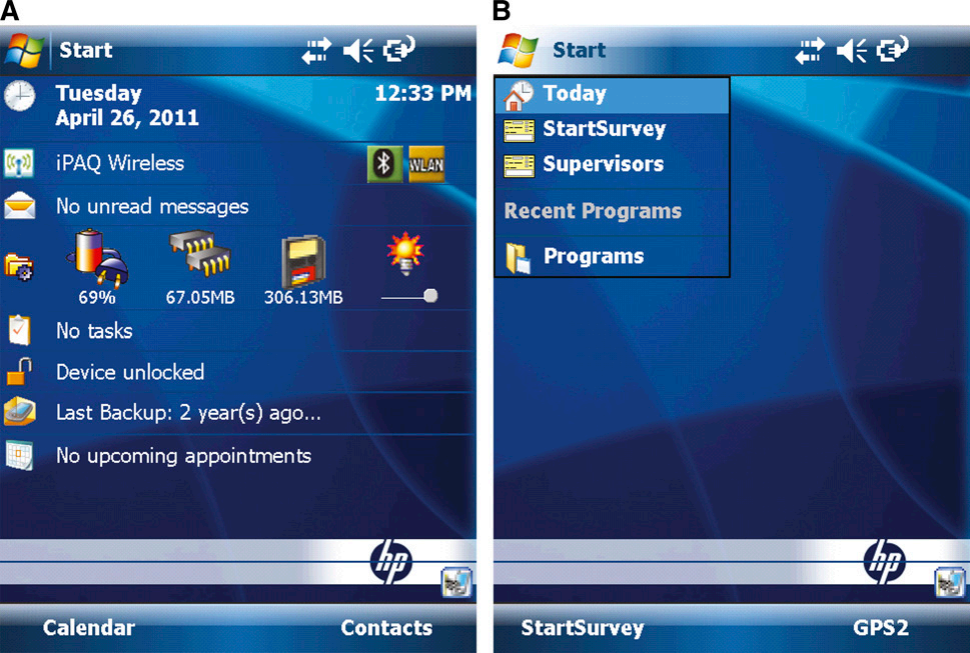
Comparison of two personal digital assistants (PDAs) main screens before and after tailoring. (**A**) Before tailoring and (**B**) after tailoring.

In the field, reliable electricity is frequently not available for charging PDAs. Therefore, we equipped our PDAs with extended life batteries. However, we find these PDAs sometimes still cannot last long enough to support surveys all day in the field, such as those conducted in Indonesia during the rainy season. To keep batteries from being drained quickly, we disabled almost all other software attached to PDAs such as Internet Explorer, Office Mobile, Games, Media Player, etc. We also turned off all wireless networks including WLAN (a wireless local area network) and Bluetooth because continually searching for network signals also consumes a lot of battery power. Only if data among PDAs needs to be merged, will Bluetooth be turned on by supervisors. Furthermore, saving battery power by disabling these functions also prevents field workers from being distracted by the applications that might increase data entry errors. In addition, we suggest that GPS units should be unplugged when they are not in use because GPS units can drain the batteries. Finally, before finishing PDA-tailoring, we use a free third-party application called SoftKeyAppletEx^31^ to change the applications linked to the softkeys on the “Today Screen” of a PDA. For example, the two softkeys are linked to two default applications, Calendar and Contacts as shown in Figure 8A, and the two applications are replaced with the entry form of PGMS and GPS2 in Figure 8B. Figure 8 displays two PDA main screens before and after tailoring.

We used the Distribution Files Wizard in Visual CE 10 to create an installation package of PGMS on one PC, and then installed PGMS on one tailored PDA by running setup.exe in the package. We provide a demonstrative installation package to readers on Sourceforge.net. The complete download address is in the Software Availability section. The installation of GPS2 is straightforward, so we do not discuss it here. Now, the tailored PDA installed with PGMS is ready for interviewers to use. The whole process, including the PDA's tailoring and multiple software installations, usually takes a person who is familiar with the whole process about 20 minutes to complete. If we need 30 PDAs for a survey and each PDA has to be prepared in this way, a person has to repeat the previous operations 30 times, which will take about 10 hours. Obviously, this manual deployment is time-consuming and prone to mistakes. In this project, we adopted the third-party software, Sprite Clone,^32^ which provides a rapid means of doing mass installation. We use Sprite Clone to clone the tailored and PGMS-installed PDA, which generates an image (a single file containing the whole system), and then we store the image on a SD card. After the SD card is plugged into other PDAs, it can automatically setup the whole system of the cloned PDA on them. The system clone usually takes 5 minutes and the image installation on a PDA only needs 3–4 minutes. Therefore, after the master PDA is prepared, the time for preparing the remaining 29 PDAs is only about 2 hours.

More details about tailoring and cloning PDAs for surveys can be found in the Supplement of Text S1.

## Results

**Figure 9.**
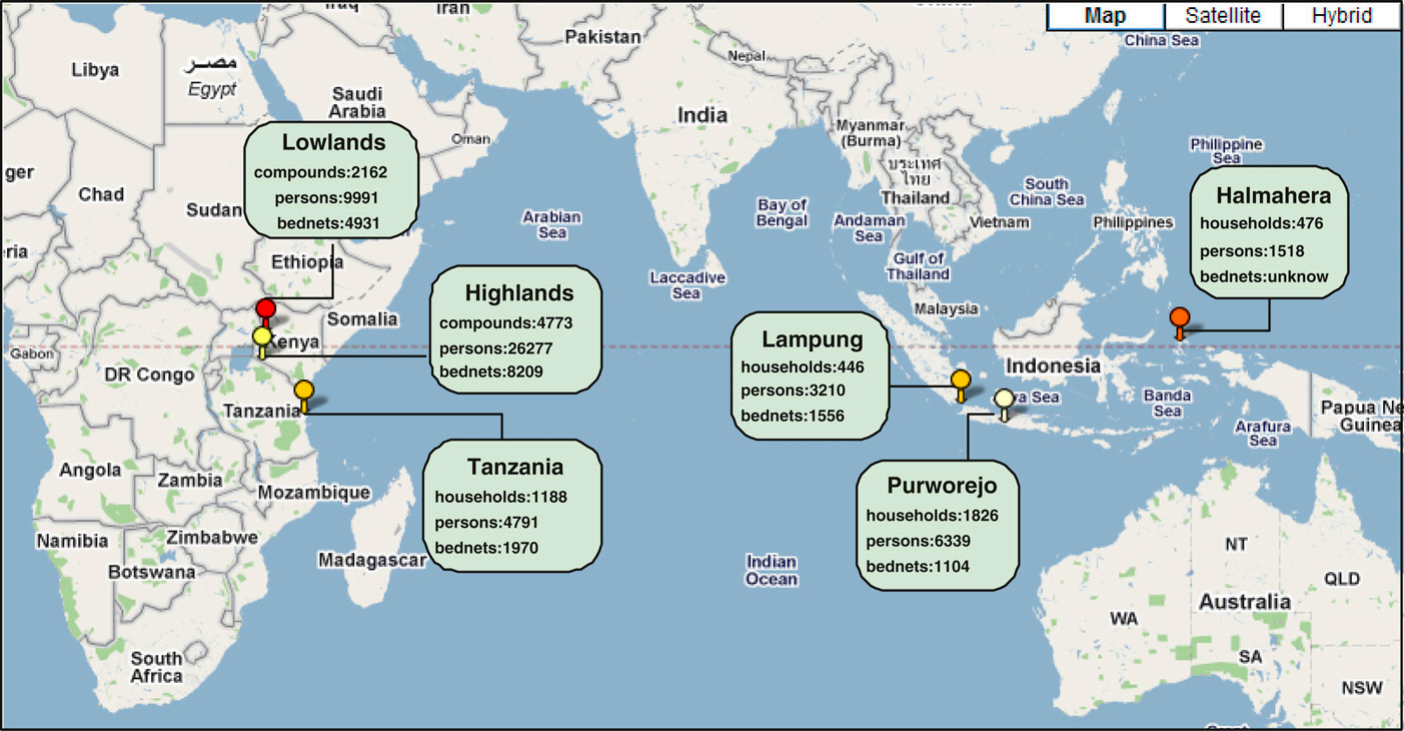
Distribution of survey data collected by personal digital assistant (PDA)-Geo-tagged malaria-related data collection tool (PGMS) from the six study sites.

Ten thousand eight hundred and seventy-one (10,871) compounds and households, 52,126 persons, and 17,100 bed nets at the six study sites across African and Asian continents have been recorded by means of PGMS since 2008. Those numbers will increase as future surveys are conducted by PGMS. All data are archived in a central MTC database at University of Notre Dame. More information about the data collected from the study sites can be found in the MTC portal.^2^ Figure 9 shows the distribution of received data among the six sites. These data are MTC-related. The not-MTC data collected by CDC in its parasitemia and anemia survey since 2010 include 868 compounds, 5,993 persons, and 2,714 bed nets (not shown in Figure 9).

**Figure 10.**
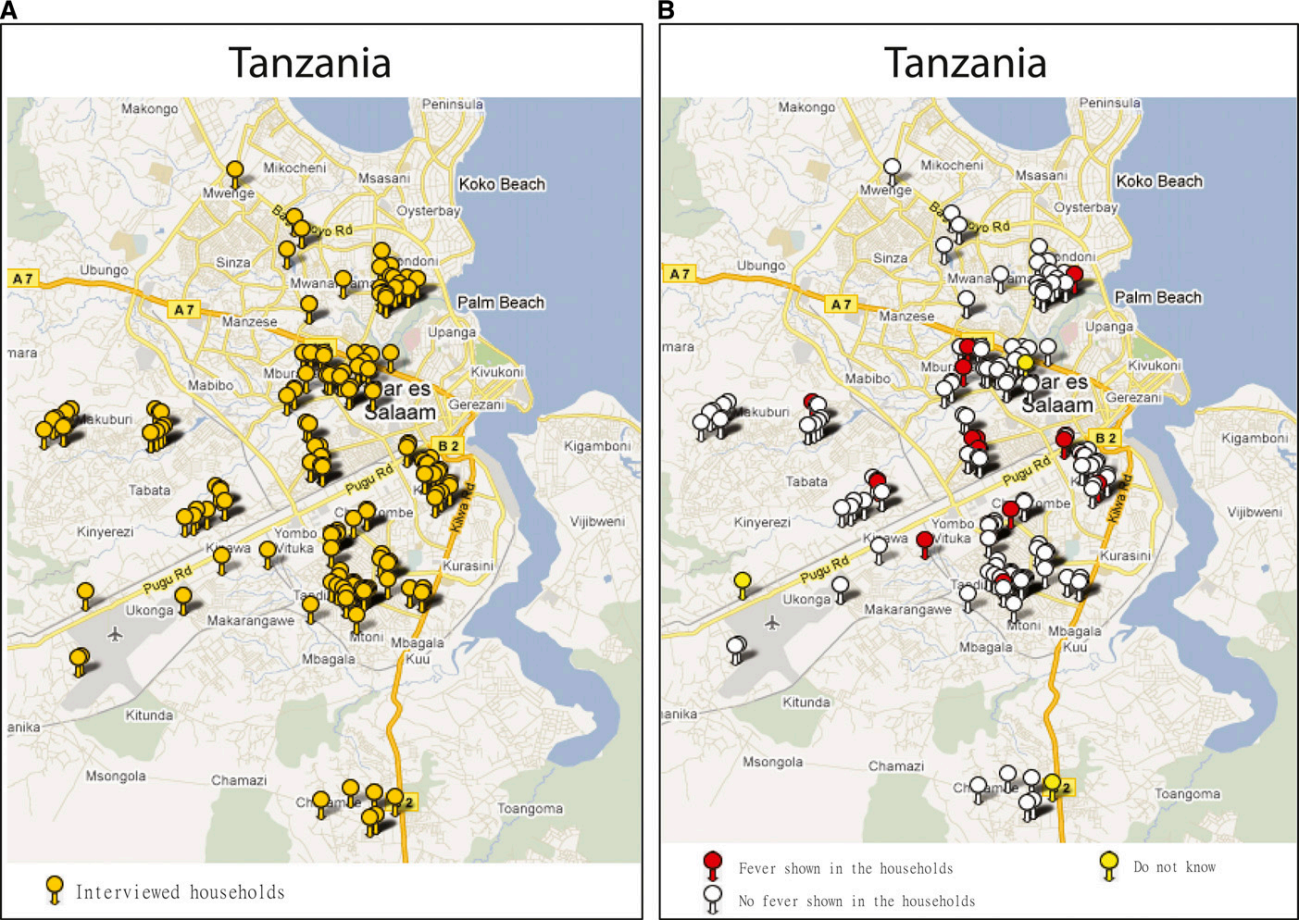
A distribution of 230 demonstrative households and fever cases reported by them. (**A**) Map of interviewed households. (**B**) Map of fever reported by interviewed households.

In this work, we present a demonstration of representative survey data to show what they look like using the Tanzania site as an example. Figure 10 presents a 230-household demonstration on the Google Map based on their coordinates exported from PDAs for the purpose of quickly reviewing the distribution of interviewed households and potential malaria cases reported by them. Figure 10A displays the 230 household points on a map provided by Google Maps. In Figure 10B, three kinds of demonstrative points are color-coded to represent the three responses to the question in the person questionnaire: Has anyone been ill with a fever at any time in the last 2 weeks in this household? (The two demonstrative figures, Figure 9 and Figure 10, are generated by spatialepidemiology.net on a personal computer.)

**Figure 11.**
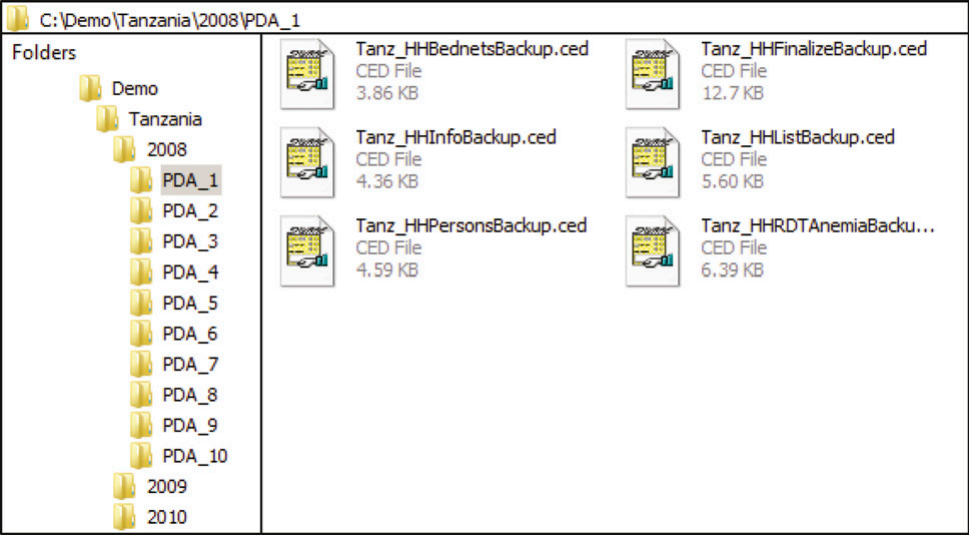
The organization of the 3-year raw data files and folders.

The collected data could be stored in the central database in two ways. One way is to store all raw data files collected at the six study sites since 2008 on a data server and organize the files as shown in Figure 11. Alternatively, we could dump the data out of these files and merge them into six Microsoft Access tables by means of the method discussed in the Survey Data Storage and Synchronization between PDAs and PCs section.

## Discussion

Although the initial investment for a PDA-based MIS questionnaire is greater than a paper-based one, many of the costs are incurred once. The PDAs we purchased have been used on multiple surveys in the field for +5 years, with 70% functional after that period. Examples of failures in the remaining 30% of the PDAs include broken connectors, batteries not holding a charge, power buttons hard to turn on, and GPS units not working. A proper life cycle cost comparison between paper-based and PDA-based surveys should include the cost of the required replacement units. On the contrary, the cost of data entry for paper-based data is incurred for each survey. This is especially true for large-scale surveys, where many questionnaire pages need to be printed, and afterwards require the repeated labor-intensive data entry activities. Moreover, for paper-based surveys, hardware such as handheld GPS receivers and computers for data entry are still needed. In the long run, the cost of hardware and software purchased at the beginning of the study and the cost of the required replacement units after each round are balanced by the cost of paper for questionnaire printing, data entry, and data cleaning with possible lower quality and reliability.

In our electronic questionnaires, each question's text can only be changed in the Visual CE development environment. Therefore, interviewers cannot switch among the languages on the fly. CSpro that also runs on Windows Mobile 5 and 6 allows each question's text to show in multiple languages at the top of the screen, and the interviewer can switch among languages during the survey. However, CSpro does not translate the text of each response. This method is similar to the first translation method described in the Translating Electronic Questionnaires in Local Languages section.

There is no guarantee that the questionnaire can be finalized well in advance of the survey because requirements often change. Therefore, the framework of PGMS should be flexible for epidemiologists, ecologists, and those who often collect data in the field to amend the electronic questionnaires without much difficulty. To reach the goal, we divide the tailored paper questionnaires into seven parts, and implement the design that any modifications within each part do not affect the whole system architecture. From the feedback of PGMS's users at the six study sites, the framework of PGMS has proven to be flexible, permitting persons with little programming experience to easily modify questionnaires in the field by adding new survey questions or deleting/editing existing questions from each electronic questionnaire in PGMS. Moreover, we also developed a few simple programming demonstrations as tutorial material to help train the local staff so that they can make basic modifications on the electronic questionnaires. In the highlands of Kenya, as many as 120 field staff were trained on the use of PDAs for the surveys. Of these about a quarter were community health workers. Some of the staff have the skills to use PDAs for other ongoing programs. In the lowlands of Kenya, around 80–100 persons were trained on the use of PDAs for data collection over the course of MTC. They were mostly nurses and community health workers who were responsible for primary data collection. Three people were trained to do downloading/trouble shooting/programming. Although, some researchers picked up a bit of PDA programming knowledge, we mostly targeted the training toward the field workers who deal with conducting surveys.

The solutions to addressing the two challenges that people might face when deploying a PDA-based survey have also been proven by field workers and survey supervisors to be effective in preventing the accidental loss of valuable field data and making data entry easier. Some unpredictable mechanical and environmental factors, such as battery failure, PDA damage caused by harsh weather, the SD card not being inserted properly, or even PDA loss, can result in missing or compromised data. In these cases, if the data were backed up to the SD cards daily, we functionally lost about a half-day of data. It involved revisiting the households and then redoing the surveys for the half-day missing data.

For the sites that already have households geo-tagged ahead of time, and thus do not need GPS2 to map all households and select a randomized sample, we have to generate the input files for their PGMSs manually or through some third-party software such as Microsoft Excel.

In addition to repeatedly emphasizing the techniques to avoid accidentally deleting records during the training session, we also adopted the method discussed in the Database Design for Cross-Sectional Data Analysis section to help prevent incomplete data or data loss that is caused by human errors. Efforts are also made to ensure data consistency in the PGMS database. For example, in the system with the compound as the sampling unit, one household record cannot be deleted unless the record(s) of the person(s) and/or the bed net(s) in this household are/is deleted first. Another example is that if the two blood testing results of an interviewee have been entered into the system, the person's information record entered on the HHPf form cannot be deleted unless the interviewer goes to the HHPRAf form to delete the interviewee's blood testing results first.

As mentioned earlier, we suggest that GPS units should be unplugged when they are not in use to minimize unnecessary battery loss. However, during the survey implementation, people reported that it is inconvenient to take GPS units out of/place them into OtterBox 3600 Cases, and also mentioned that the cases are clumsy. As a result, one study site decided to stop using the cases and chose an alternative: plastic over-the-arm wallets available locally that have zips so that they can easily take PDAs and GPS units out of/place them into the containers. To solve this problem in a future survey, we might need to replace the compact GPS units with those that would not drain the battery as quickly and adopt lightweight cases.

The disadvantages of PDAs cannot be ignored. Some problems with the PDAs have been reported to us from our studying sites. For example, we found a Hewlett-Packard iPAQ 210 Series PDA with 128 MB main memory for running applications is only able to store at most around 1000 records so that the PDA memory has to be cleared after about 1000 records have been input, otherwise the PDA might crash. Although we have taken measures for preventing battery power loss with the PDAs, daily charging at most of the sites is still required. Therefore, portable solar panel chargers are usually needed. In addition, all sites need technicians who are trained on PDA maintenance/fixing.

The Visual CE Pro Edition we purchased allows their runtime Visual CE system, and the other redistributable components that PGMS needs to run to as many users as we want. Namely, Visual CE Pro Edition has a royalty-free runtime system.

As Microsoft announced that Windows Phone is not compatible with Windows Mobile devices, and will supersede Windows Mobile, suitable mobile handhelds might be difficult to find in the future market. If this happens, our programs will need to be migrated to Android by DroidDB. So far, we have not found migration paths for our software to other mobile operating systems such as iOS.

In this work, we mainly show the methodology of compound/household-based electronic questionnaire and database development and the experience and lessons we learned from our survey deployment on the six study sites. Therefore, we would like to present more high-level developing ideas here, rather than any specific Visual CE code. Equipped with these ideas, sometimes rewriting the code might be easier than trying to find ways to migrate the code to any specific platforms.

Using mobile devices, such as PDAs, smartphones, pocket PCs, etc., to electronically collect data is the way for the field questionnaires in the future. The major point of this work is not to prove our software is the best solution and Visual CE is the only option. We just would like to present a complete case study including PGMS, the third-party software, and PDAs running Windows Mobile we adopted in our project. With them, we have successfully accomplished a few MTC surveys across the six field sites between 2008 and 2012. Except for Visual CE, there are a few other electronic-questionnaire development tool candidates such as Visual Studio.Net, CSPro, Open Data Kit Collect,^33^ Droid DB, and so on. Except for PDAs running Windows Mobile, smartphones, tablet computers, and other portable digital devices having iOS, Android, Windows Phone, WebOS, etc., are also good candidates that can be considered when implementing large-scale surveys in the field.

## Software Availability

All forms described in this work and a test input file called Location.txt are stored in an open software archive. You need to purchase Visual CE Personal or Professional to open or edit these forms. If you just want to run PGMS on your Windows Mobile PDAs, you do not need to purchase Visual CE. Visual CE Professional Version provides royalty-free runtime programs. The source code and the PGMS runtime installation package can be downloaded for free at: http://sourceforge.net/projects/mtcpgms/.

## Supporting Information

Text S1: Preparing PDAs of HP iPAQ 210 Series for MTC surveys. (707 KB PDF).

Text S2: How to dump data from PDAs to a Microsoft Access database. (28 KB PDF).

Text S3: Glossary. (9 KB PDF).

Text S4: List of hardware and software. (9 KB PDF).

Text S5: Relationships among the tables. (40 KB PDF).

Excel E1: The description of the fields in each data table. (27 KB XLS).

Multimedia S1: How to conduct a complete MTC survey by using PGMS. (9.12 MB AVI) These files can be downloaded from https://sourceforge.net/projects/mtcpgms/files/SupportingFiles/.
